# Successful Use of Cytokine Hemadsorption Filter In Montivipera xanthina Envenomation: A Case Report

**DOI:** 10.7759/cureus.77807

**Published:** 2025-01-22

**Authors:** Ahmet M Savas, Yunus E Ozluer, Onur C Buyuktas, Faruk Akkus

**Affiliations:** 1 Emergency Medicine, Aydin Adnan Menderes University, Aydin, TUR; 2 Emergency Medicine, Erzurum City Hospital, Erzurum, TUR

**Keywords:** hemadsorbtion, inflammation, perfusion index, snake envenomation, toxicology and envenomation

## Abstract

Severe local and systemic effects can occur after *Montivipera xanthina* envenomation. In this case report, we demonstrate the successful use of CytoSorb^®^ (CytoSorbents, Princeton, New Jersey) hemoadsorption filter to remove snake venom from the systemic circulation in a patient with severe local toxicity after *Montivipera xanthina* envenomation. A 62-year-old woman was bitten on the fifth finger of her left hand by a viper snake of the genus *Montivipera xanthina*. She complained of numbness in the left arm, chest pain, and mild shortness of breath. There was a double tooth mark, severe edema, and necrotic appearance on the fifth finger of the left hand. Since it was learned that there would be a delay in the supply of antivenom, CytoSorb^®^ hemoadsorption therapy was administered to the patient with signs of severe local circulatory disturbance. Since the beginning of the treatment, the patient's tissue perfusion improved significantly. Subsequently, antivenom treatment was also provided and a significant improvement in necrotic tissue appearance and edema was observed in the follow-up. This case demonstrates that early use of hemoadsorption therapy can be effective in snakebite cases with systemic and local clinical manifestations. It is the first case in the literature in which CytoSorb^®^ filter successfully removed snake venom.

## Introduction

Snake bites are among the most common environmental emergencies, especially in tropical climates, and cause severe local and systemic effects caused by complex venoms that can rapidly enter the systemic circulation [1,2]. Snake venoms consist of peptides and proteins with molecular weights of 6-350 kDa and that are composed of pharmacologically active enzymes, such as metalloproteinases, phospholipase A2, and serine proteases [3]. The peptides of the venom of *Montivipera xanthina* (banded viper), a member of Palearctic vipers and an endemic species in western Türkiye and Greece, have a molecular weight of up to 9 kDa [4]. Hypotension may develop due to various venom activities, as well as due to permeability factors that may also cause hypovolemia as a result of extravasation of plasma and toxins that affect cardiac muscle, vascular smooth muscle, and other tissues. Some local myotoxic and cytotoxic effects also cause local tissue necrosis [5]. These effects may result in tissue edema, vasoconstriction, and capillary endothelial damage, which, in rare cases, may lead to compartment syndrome [6]. Administering antivenom is the mainstay of treatment after a snake bite. At times, however, the antivenom may be inaccessible for several reasons. Alternative treatment methods are limited when antivenom is not available.

The perfusion index (PI) is a non-invasive measure of the ratio of peripheral pulsatile arterial blood flow to non-pulsatile blood flow. It is derived from the photoplethysmography signal. In healthy individuals, PI values can range from 1% to 10%. Low PI values usually indicate poor peripheral circulation, which is a sign of insufficient tissue perfusion, and high PI values are usually associated with increased tissue perfusion and peripheral circulation, such as inflammation. This may also occur due to vasodilatation or the pharmacological effects of certain medications [7].

Increased cytokine release due to the effects of snake venom intensifies the severity of symptoms [8]. Cytokine adsorption is a treatment method that regulates the immune response after targeting cytokines with the hemadsorption method and helps prevent multiple organ failure. CytoSorb® (CytoSorbents, Princeton, New Jersey) is a whole-blood filter that consists of porous adsorbent beads formed by crosslinking biocompatible polystyrene with divinylbenzene and polyvinylpyrrolidone [9]. Filtration with this extracorporeal cytokine adsorption device removes inflammatory mediators and endogenous molecules with molecular weight ranges of 5-60 kDa [10].

This case report is on the successful use of CytoSorb® to treat a patient bitten by a *Montivipera xanthin*a.

## Case presentation

A 62-year-old woman with a known history of hypertension, hypothyroidism, and coronary artery disease was admitted to a tertiary hospital emergency department approximately two hours after she was bitten by a viper on the fifth finger of her left hand. Upon admission, she reported numbness and pain in her left arm, chest pain, and mild dyspnea. A physical examination revealed a double fang mark, swelling, pain, ecchymosis in the fifth finger of her left hand, and edema extending from her left arm to below her elbow. Her peripheral pulses were patent. The vital signs of the patient upon admission were as follows: blood pressure 146/87 mmHg, heart rate 63/min, respiratory rate 16/min, temperature 36.6°C, and oxygen saturation 97%. The patient showed a photograph of the snake taken at the time of the incident, and based on its distinctive features, the snake species was identified as *Montivipera xanthina*. Since the antivenom was not readily available at our institution, we elevated the affected limb and provided analgesia (intravenous 1 g paracetamol and 100 mg tramadol) to reduce local inflammation and control symptoms while reporting the incident to the Provincial Health Directorate so that they could obtain snake antivenom for the patient. Tetanus prophylaxis was administered to prevent tetanus infection. In addition, intravenous cefazolin (2 g) and gentamicin (320 mg) antibiotics were given prophylactically to prevent secondary infections.

**Figure 1:**
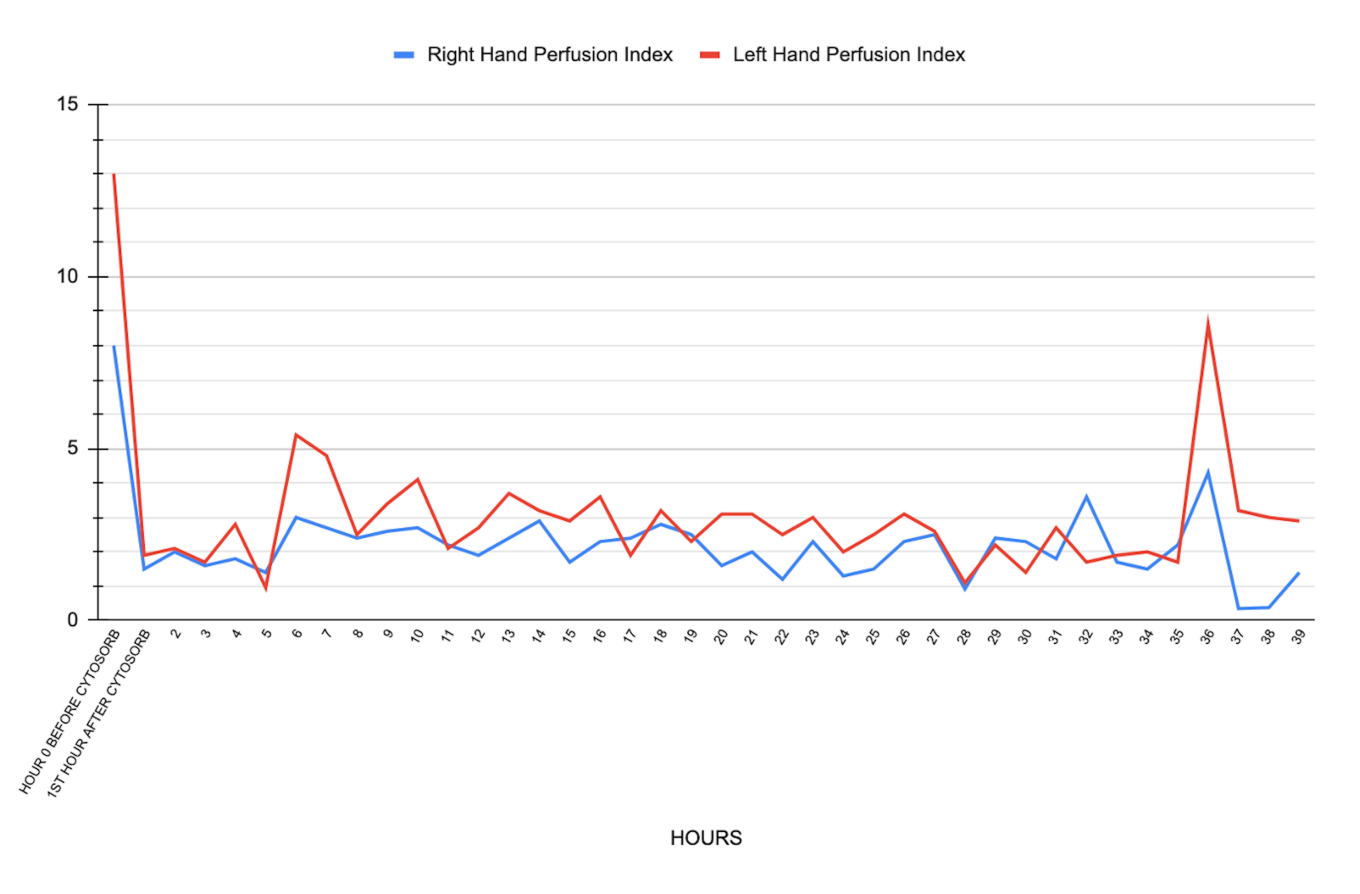
Perfusion index monitoring of both upper extremities before and during CytoSorb® treatment

**Figure 2:**
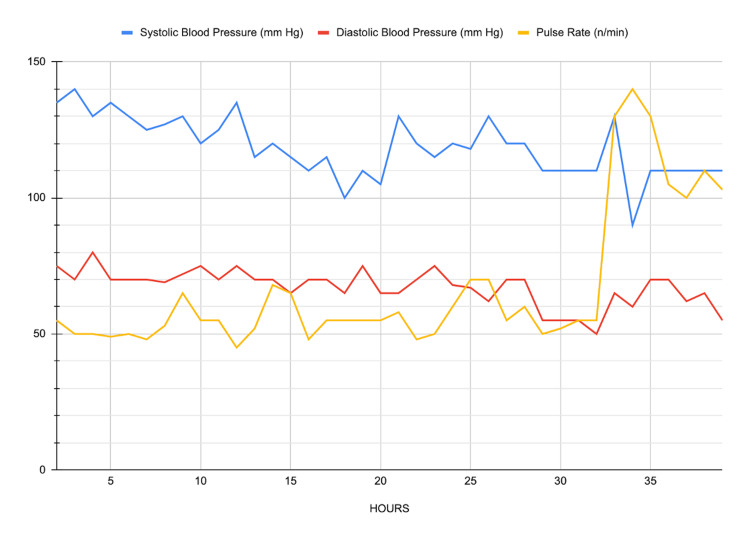
Follow-up of vital signs before and during CytoSorb® treatment

Initial laboratory tests, including complete blood count, renal function, electrolytes, and coagulation parameters, were performed, and the results were as follows: platelet count 118.000/μL, hemoglobin 12 g/dL, urea 29 mg/dL, creatinine 0.71 mg/dL, INR 0.97, aPTT 28.3 seconds, fibrinogen 219 mg/dL and D-dimer 575 ng/mL. Electrolytes and other parameters were within normal limits. Due to the delay in obtaining antivenom, circulatory disturbances and necrosis of the fifth digit worsened, requiring additional interventions. Twelve hours after the patient's admission, signs of circulatory disturbance and necrotic appearance increased in the fifth finger of her left hand, and a fasciotomy was performed. However, there was no clinical response in terms of the reduction of tissue edema or circulatory disturbance. The PI was measured from the second finger of both hands to monitor the peripheral circulation (Figure 1). Due to the continued delay in the supply of antivenom, a right jugular central venous catheter was placed, and CytoSorb® treatment was started with a continuous renal replacement therapy device. In the first 24 hours, the filter was changed once every 12 hours and once on the following day. The total filtration time was 39 hours. The trend of the vital signs of the patient during the CytoSorb® therapy is shown in Figure 2. The snake antivenom was obtained five hours after the commencement of the CytoSorb® treatment, and three vials of it were administered to the patient, after which the patient's PI values remained stable within normal limits. On the following day, the necrotic appearance and edema in the left hand decreased significantly, and the patient showed clinical improvement. İnformed consent was obtained from the patient for participation in the study.

## Discussion

This case demonstrated successful hemofiltration of viper venom with CytoSorb® to manage impaired systemic and tissue perfusion after a viper bite. The circulatory disturbance and local tissue necrosis that developed in the patient were the systemic and local effects of snake venom and are compatible with the inflammatory response and microvascular damage reported in the literature [10]. In this case, PI was used as an important parameter for monitoring the peripheral circulation and the follow-up of the treatment findings. The high PI values before the treatment indicated local vasodilatation and edema due to inflammation, whereas the decrease in the PI values after the initiation of the CytoSorb® treatment indicated that the circulation had stabilized. We also think that the high PI value measured from the contralateral upper extremity before the hemadsorption was related to the systemic inflammation caused by the snake toxin. The dramatic decrease in the PI values measured from the unaffected upper extremity may reflect the effectiveness of CytoSorb® treatment in suppressing systemic inflammation.

The snake antivenom (Polisera Snake Antivenom, Vetal Sera and Biological Products Production and Marketing Co., Adıyaman, Türkiye) used in Türkiye has a molecular weight of 100-110 kDa [11]. The delay in administering antivenom to the patient due to the initial unavailability of antivenom due to a temporary shortage in local hospital and provincial stocks necessitated consideration of alternative treatment modalities. Since the molecular weight of the venom was within the filtration range of CytoSorb® and the molecular weight of the antivenom was outside this range, we hypothesized that CytoSorb® may theoretically filter inflammatory mediators and snake venom during treatment and remove them from the circulation while allowing the snake antivenom to remain in the circulation. CytoSorb® treatment was found to contribute to the reduction of tissue edema and the suppression of systemic inflammation, as seen in the PI follow-up, in addition to the restoration of the tissue perfusion to normal levels.

Paul et al. also used hemadsorption to manage cytokine storms in patients who had developed medical conditions such as sepsis, septic shock, multiple organ failure, and gangrene after a snakebite [12]. However, to our knowledge, our case is the first case in literature in which a hemadsorption filter was successfully used only to remove venom from the blood. In addition, prompt CytoSorb® treatment might have positively affected the prognosis of envenomation in the early period and might have prevented the occurrence of systemic effects of envenomation.

The most important limitation of this case report is that the effect of hemadsorption was evaluated with indirect data, such as clinical findings and PI since we were unable to measure the venom level.

## Conclusions

This case report shows that treatment with the CytoSorb^®^ hemoadsorption method may be useful in the management of tissue damage after viper snake bite in addition to antivenom treatment in the early period. With this treatment method, inflammation is reduced, and snake venom is removed from the circulation. It is thought that the snake antivenom given can remain in circulation without being filtered because it is in the range of 100-110 kDa. This method of treatment can be particularly effective in snake envenomations with a molecular weight within the filtration range of the hemoadsorption filter and in cases with severe systemic inflammation, circulatory disorders, or significant tissue damage. Significant improvement was observed in the patient's symptoms after treatment. However, more large-scale studies are needed for the generalizability of these findings.
